# The Effects of Pro-Inflammatory and Anti-Inflammatory Agents for the Suppression of Intimal Hyperplasia: An Evidence-Based Review

**DOI:** 10.3390/ijerph17217825

**Published:** 2020-10-26

**Authors:** Rohaina Che Man, Nadiah Sulaiman, Mohamad Fikeri Ishak, Ruszymah Bt Hj Idrus, Mohd Ramzisham Abdul Rahman, Muhammad Dain Yazid

**Affiliations:** 1 Centre for Tissue Engineering & Regenerative Medicine, Universiti Kebangsaan Malaysia Medical Centre, Jalan Yaacob Latif, Cheras 56000, Kuala Lumpur, Malaysia; enaroha@yahoo.com (R.C.M.); nadiahsulaiman@ukm.edu.my (N.S.); fikeri_ishak@yahoo.com (M.F.I.); ruszyidrus@gmail.com (R.B.H.I.); 2 Department of Physiology, Faculty of Medicine, Universiti Kebangsaan Malaysia Medical Centre, Jalan Yaacob Latif, Cheras 56000, Kuala Lumpur, Malaysia; 3 Department of Surgery, Faculty of Medicine, Universiti Kebangsaan Malaysia Medical Centre, Jalan Yaacob Latif, Cheras 56000, Kuala Lumpur, Malaysia; drramzi@ymail.com

**Keywords:** cardiovascular disease, chemokines, cytokines, intimal hyperplasia, neointimal hyperplasia, atherosclerosis

## Abstract

Anti-atherogenic therapy is crucial in halting the progression of inflammation-induced intimal hyperplasia. The aim of this concise review was to methodically assess the recent findings of the different approaches, mainly on the recruitment of chemokines and/or cytokine and its effects in combating the intimal hyperplasia caused by various risk factors. Pubmed and Scopus databases were searched, followed by article selection based on pre-set inclusion and exclusion criteria. The combination of keywords used were monocyte chemoattractant protein-1 OR MCP-1 OR TNF-alpha OR TNF-α AND hyperplasia OR intimal hyperplasia OR neointimal hyperplasia AND in vitro. These keywords combination was incorporated in the study and had successfully identified 77 articles, with 22 articles were acquired from Pubmed, whereas 55 articles were obtained from Scopus. However, after title screening, only twelve articles meet the requirements of defined inclusion criteria. We classified the data into 4 different approaches, i.e., utilisation of natural product, genetic manipulation and protein inhibition, targeted drugs in clinical setting, and chemokine and cytokines induction. Most of the articles are working on genetic manipulation targeted on specific pathway to inhibit the pro-inflammatory factors expression. We also found that the utilisation of chemokine- and cytokine-related treatments are emerging throughout the years. However, there is no study utilising the combination of approaches that might give a better outcome in combating intimal hyperplasia. Hopefully, this concise review will provide an insight regarding the usage of different novel approaches in halting the progression of intimal hyperplasia, which serves as a key factor for the development of atherosclerosis in cardiovascular disease.

## 1. Introduction

### 1.1. Vascular Intimal Hyperplasia, Risk Factors and Current Therapy

Cardiovascular disease (CVD) is a primary cause of death for certain regions in the world, which can be a great hindrance to a continuity of human life [1]. Atherosclerosis is recognized as a major leading cause for CVD [2,3] causing obstruction of the vessels, through soft plaque calcification [4], which hardens, thickens and narrows the inner wall arteries [5,6]. Atherosclerosis progresses into coronary artery disease, causing the shortage or reduction of oxygen-rich blood flow [7] to the cardiac muscle. This condition will eventually contribute to critical cardiac problems, including myocardial infarction [8] and chronic cardiac failure [9]. Intimal thickening is considered as a precursor to lesion progression in atherosclerosis, but the primary factors contributing to its emergence remain elusive. In general, atherogenesis can be divided into several key phases [10], which include the endothelial dysfunction or impairment, generation of lipid within the intimal region, cell growth and migration of smooth muscle cells and leukocytes across the vascular wall, formation of foam cell and deterioration of extracellular matrix [11].

Intimal hyperplasia (IH) is an abnormal cell aggregation that has been observed in various developments of vascular diseases [12], mainly in atherosclerosis, occlusion of veins [13] and synthetic vascular grafts, in-stent restenosis and coronary angioplasty [14]. This process is closely related to the increment of cell numbers [15,16]; thus, its formation is frequently associated with vascular cell activation [17]. There are a few types of cells that are linked to the development and continuation of this process, predominantly vascular smooth muscle cells (VSMCs) [18], vascular adventitial fibroblast cells [19], vascular endothelial progenitor cells [20] and bone marrow derived progenitor cells [21], which originated from the different lining. VSMCs is crucial for the progression of intimal stiffening and development of neointimal hyperplasia [22], via cell migration from the media to intima layer and deposition of increased extracellular matrix, which finally comprise about 60–80% of the intimal region [23]. This may result in high cellular density within the lesion in the subintimal area, eventually decreasing the size of the lumen and contributing to thrombosis in the blood vessels [24].

Recent studies reported the numerous risk factors promoting the development of IH. Most of the main risk factors for IH are due to vascular wall injury, especially in the event of balloon angioplasty [25] and stents positioning [26]. These events remarkably exacerbate both thrombosis and increase the rate of IH [27], infiltration of inflammatory cells that regulate inflammatory response, which play a crucial role in intimal thickening after coronary stenting [28] and ageing of human arteries [29].

Over the past few decades, various interventions performed by the clinicians to circumvent atherosclerosis, particularly by percutaneous transluminal angioplasty (PTA) [30], placement of medical devices such as stents [31] or drug-eluting stents [32], the use of human saphenous vein (HSV) grafts in coronary artery bypass grafting (CABG) surgery [33] or peripheral bypass surgery [34] and coronary endarterectomy [35]. In spite of the numerous advantages, these interventions remained unsuccessful in preventing arterial restenosis caused by neointimal hyperplasia [36,37]. To date, an effective strategy to counteract this unsatisfactory outcome is yet to be discovered. This review article will explicate the essential values of different factors and their significant effects towards an alternative intervention in combating IH development.

### 1.2. Chemokines and/or Cytokines Effects in Inhibiting Intimal Hyperplasia Development

Active involvement of chemokines and/or cytokines is mostly connected with diverse biological processes. These processes are primarily consisting of cellular attachment, proliferation, migration, differentiation, growth, maintenance and signal transduction, inter and intracellular communication and activation of various immune response. In recent years, several reports suggested that selecting chemokines with the combination of cytokines may have a promising effect on halting the progression of IH. A wide range of anti-inflammatory or pro-inflammatory cytokines and/or chemokines has been studied and proven to gradually decrease the inflammatory events in the IH and atherosclerosis.

Atherosclerotic vascular disease pathological event involves inflammatory response, which promotes macrophages and lymphocytes infiltration into the vascular wall. The mediators contributing to this process are normally chemokines and their receptors [38]. Monocyte chemoattractant protein-1 (MCP-1) is expressed by macrophages, endothelial cells (ECs) and vascular smooth muscle cells (VSMCs) [39]. It is a member of the C-C chemokine family and has a great impact on the migration of monocytes or macrophages that contributes to inflammatory response in atherogenesis. Monocyte chemoattractant protein (MCP)-1 is one of the pro-inflammatory chemokine that is positively expressed in atherosclerotic plaque and in the intimal area during the arterial injury. CC chemokine receptor 2 (CCR2) is recognized as a functional receptor for MCP-1. Roque et al. (2002) [40] showed that CCR2 has a significant role in affecting the growth of smooth muscle cell and the progression of IH in an animal model of chronic arterial injury. This study had also proved that infiltration of macrophages was not significant, when rapid adherence of leukocytes to the arterial surface injury are maintained. On the contrary, Furukawa and co-workers (1999) [41] demonstrated that the use of anti–MCP-1 treatment significantly attenuated the development of neointimal hyperplasia, before and immediately after the carotid arterial injury. These findings can further clarify the IH inhibitory effects of the MCP-1 in vivo.

Another one of such pro-inflammatory cytokines that is secreted by macrophages/monocytes upon severe inflammation is tumour necrosis factor-alpha (TNF-α). TNF-α plays a key role in a various cell signalling events [42] and is recognized as one of the many cytokines that significantly upregulated in various diseases. It is also thought to be as one of the pathological factors for a number of common arterial diseases, such as atherosclerosis and preeclamptic hypertension [43,44]. The most recent study by Maleknia and colleagues (2020) [45] reported that TNF-α and interleukin-1 (IL-1) in the early inflammation decreased IH leading to restenosis, by inducing the production of insulin-like growth factor binding proteins (IGFBPs) in the vascular wall.

Chemokines such as MCP-1 are potent chemotactic agents for monocytes [46]. The cytokines such as pleiotrophin and TNF-α were recognized as key factors in promoting trans differentiation of monocytic cells to endothelial-like cells [47]. Based on the significant effects of chemokines/cytokines, we suggest that they could be used with combination of the scaffold to inhibit the progression of intimal hyperplasia. In the future, the coating of scaffold with these chemokines and cytokines may therefore facilitate the attraction of monocytes to the scaffold and induce differentiation into endothelial-like cells to line the scaffold and attenuate the thrombus formation, intimal thickening and beneficial remodelling. Moreover, the scaffold will induce in situ cell recruitment and differentiation [48], which will avoid the need for ex vivo tissue engineering, which is costly and time-consuming. Thus, we suggest that future modification using coated chemokines/cytokines to the scaffold is required to improve the graft performance and this novelty could minimize the operating time, clinical risks, and expenses.

Therefore, the synergistic effect of the proinflammatory cytokines and growth factors in the pathologic event of restenosis can be considered as an early promising factor for future application of alternative cardiac therapies. In this short review, we concentrate on recent novel discoveries using anti-inflammatory and pro-inflammatory chemokines and cytokines as a therapeutic agent, particularly in the suppression of IH, restenosis and atherosclerosis.

## 2. Methods

### 2.1. Search Strategy in Selected Databases

The review procedures were methodically carried out by screening all published journal articles that are related to the effects of using different chemokines and cytokines for cardiovascular disease therapy. There are two separate databases that were systematically used to explore the related study, which are Pubmed and Scopus. The combination of keywords used were monocyte chemoattractant protein-1 OR MCP-1 OR TNF-alpha OR TNF-α AND hyperplasia OR intimal hyperplasia OR neointimal hyperplasia AND in vitro.

### 2.2. Criteria of Selection

Studies published in English within a 5-year limit ranging from 2015 to 2020 have been reviewed. Only published articles that provide free full text articles were considered. Titles and abstracts that fill the topic requirements were systematically screened. All research articles related to the effect of cytokines and chemokines for the treatment of cardiovascular disease were included. The selection criteria exclude all case reports, clinical trials, systematic reviews, letters, technical reports and editorial publications.

### 2.3. Management of Data Extraction Table

Data from selected articles were extracted by two reviewers. The articles selection underwent several screening processes before being included in the data extraction table. Titles were carefully screened to meet the requirement of the topic of interest. Next, unrelated articles were omitted based on the abstracts of the shortlisted articles. At the end of the process, all identical papers were removed. Extracted information were outlined in a data extraction table as follows: (1) authors, (2) type of cells, (3) type of cytokines/chemokines involved, (4) type of disease, (5) methodology, (6) results and (8) conclusions.

## 3. Results

### 3.1. Search Results

**Figure 1:**
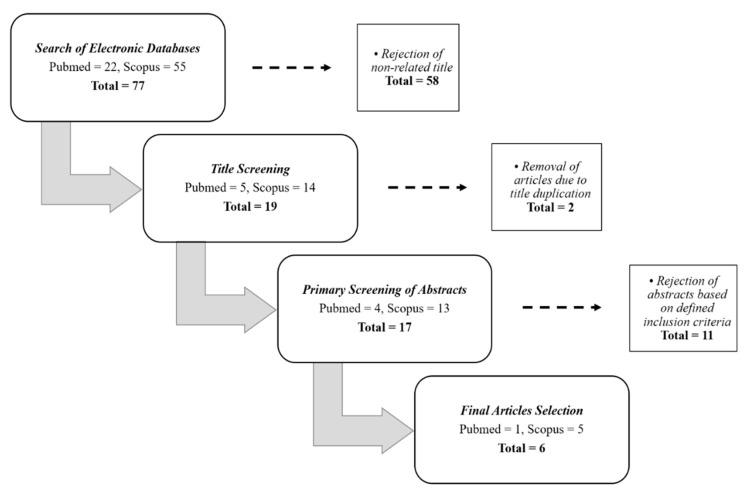
The process flow of the final articles selection from both Pubmed and Scopus databases.

The delineated inclusion and exclusion criteria of selected articles were separately evaluated by two reviewers to ensure objectivity of final articles selection. This was followed by discussion between authors to establish a consensus in assessing the differences surfaced upon articles assessment. The combination of keywords used during the search process successfully identified 22 articles acquired from Pubmed, whereas 55 articles were obtained from Scopus database. From that, 17 articles from Pubmed and 41 articles from Scopus were excluded after thorough title screening, followed by the removal of 2 duplicate articles, and thus, we were left with 17 articles to work with. At the end of the review process, 11 articles were removed from the final article selection, as they were unrelated to cardiovascular disease. Therefore, only six related articles were included in the data extraction table. The article selection process is summarized in Figure 1.

### 3.2. Study Characteristics

**Figure 2:**
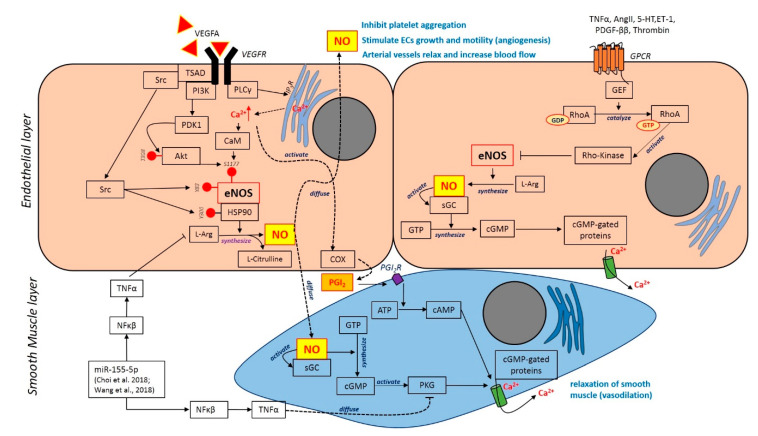
Targeted signalling pathway involved in endothelial cells (ECs) activation and vascular smooth muscle cells (VSMCs) proliferation and migration during intimal hyperplasia. The binding of vascular endothelial growth factor A (VEGFA) to its receptor VEGFR2 on ECs activates the PI3K pathway. Activated PDK1 phosphorylates Akt at Thr308 then phosphorylates eNOS at Ser1177 activating eNOS. VEGFA aslo activates the PLCγ pathway which increase Ca^2+^ concentration and activates eNOS via calmodulin (CaM). The binding of VEGFA also activates Src kinase which then activates eNOS through two mechanisms: (1) phosphorylation of Tyr83 on eNOS; (2) phosphorylation of heat shock protein 90 (HSP90) at Tyr300. Those phosphorylation causes the binding of HSP90 to eNOS and activates NO synthesis. Activated eNOS converts L-arginine in the presence of oxygen to L-citrulline and diffusible signalling molecule NO. NO secreted by ECs inhibits platelet aggregation to avoid thrombosis. NO diffuses into vessel walls, causing arterial vessels to relax and increase blood flow. The binding of agonist to G-protein–coupled receptors (GPCR) induces activation of Rho-kinase activity via GEF activation. The activity of RhoA is controlled by the guanine nucleotide exchange factors (GEFs) that catalyse the exchange of GDP for GTP. Rho-kinase activity is enhanced by binding to the active GTP-bound RhoA. However, this mechanism is negatively regulating eNOS activation for NO production. Diffused NO binds to its intracellular receptor soluble GC (sGC) which produces cGMP from GTP. The increase in intracellular cGMP concentration bind to cGMP-dependent protein kinases (PKGs) and cGMP-gated ion channels results the increase of Ca^2+^ and relaxation of smooth muscle.

In this review, the search that was conducted through Pubmed and Scopus databases identifies six related articles, which are related to intimal hyperplasia, atherosclerosis and cardiovascular disease, whereas the rest of the studies were related to hypertension and stroke. From those articles, there are several types of cells that were used in the experiment, which are the vascular smooth muscle cells (VSMC), human umbilical vein endothelial cells (HUVECs), human saphenous vein cell lines, HeLa cell lines, human micro-vascular endothelial cells (HMEC-1) bone-marrow derived macrophages, adipocytes derived mesenchymal stem cells, peripheral blood mononuclear cells (MNCs), somatic cell nuclear transfer (SCNT) and the whole blood-derived leukocytes. Most of these cells are primarily acquired from human, pig and rat samples. All of these studies also reported the use of different agents, such as cytokine, i.e., tumour necrosis factor alpha (TNF-α) and interleukin 1 beta (IL-1b); transcriptional factor or coactivator, i.e., nuclear factor kappa beta (NF-κB) and P300/CBP-associated factor (PCAF); growth factor or chemokine, i.e., platelet-derived growth factor (PDGF-BB); extracellular matrix protein, i.e., microfibrillar-associated protein 4 (MFAP4); homoisoflavonoid or compound from plant extract, i.e., 7-O-methylpunctatin (MP) extracted from bulbs of *Bellevalia eigii* (herbaceous plants), hypaphorine (Hy) extracted from *Erythrina velutina* (leguminous tree), garcinol extracted from *Garcinia indica* fruit rind (mangosteen), ganoderma triterpenoids (GT) extracted from *Ganoderma lucidum* (mushroom) and antidiabetic drug or insulin-sensitizing agents, i.e., rosiglitazone (ROS) and pioglitazone (PGZ) that were categorized in the thiazolidinedione class. Meanwhile, the other studies used the collagen gel and collagen external scaffold as part of the treatment. These factors are recognized to possess anti-atherogenic effects, thus may play a major role in preventing intimal hyperplasia caused by inflammation. Three studies conducted both in vitro and in vivo study, while the rest focused on only in vitro studies. Ultimately, the selected articles have shown that the uses of different agents, particularly the chemokines and/or cytokines, are able to promote remarkable effects in preventing intimal hyperplasia. All articles were summarized in Table 1 and Table 2. We also provide a schematic on targeted signalling pathway involved in endothelial cells (ECs) activation and vascular smooth muscle cells (VSMCs) proliferation and migration during intimal hyperplasia in Figure 2. 

## 4. Discussion

For the past few decades, inflammation has been perceived as a pivotal body defence mechanism. However, a consistent escalation of certain proinflammatory factors leads to acute inflammation, thus creating an effect called hypercytokinemia or cytokine storms. Hypercytokinemia occurs in any part of the human body and affects the internal organs, including the human heart, which is predominantly due to atherosclerosis that triggers the formation of intimal hyperplasia at the early stage [61,62,63]. Inflammation within the vascular wall is a hurdle, and manipulating the inflammatory cytokines and chemokines is possibly the key piece in solving the puzzle. Other than that, research related to inhibition of intimal hyperplasia has also been extensively done using various approaches, which utilized gene therapy and vector-mediated gene delivery [49,50,51,52,53,54,55,56,57,58,59,60,61,62,63,64], anti-inflammatory and anti-proliferative drugs [57], compounds with anti-inflammatory actions such as plants [55,65], herbal medications [50], marine sources [52] and polyunsaturated fatty acids [56].

### 4.1. Utilisation of Natural Product to Combat IH

A novel approach in mitigating intimal hyperplasia includes the recruitments of compound from plant, herbal medicine and marine sources. A study reported by Hsu and co-workers [55] suggested the efficacy of atheroprotective properties in Ganoderma triterpenoids (GT), a ganoderma mushroom, which was proven to play a significant role in preventing atherogenesis, via the elimination of disturbed flow-induced oxidative stress and inflammation. They reported that GT extract inhibited the induction of a series of atherogenic factors, including endothelin-1, von Willebrand factor and MCP-1 in carotid-artery-ligation mouse model.

A previous study by Sotokawauchi and colleagues [65] postulated that the supplementation of sulforaphane-rich broccoli sprout extract has beneficial effects in exhibiting anti-inflammatory actions. They reported that the extract reduced the gene expression of MCP-1, intercellular adhesion molecule-1 (ICAM-1), while increased the endothelial nitric oxide synthase (eNOS) mRNA levels in HUVECs. However, the active component in sulforaphane-rich broccoli sprout extract needs to be further analysed along with in vivo and clinical studies. Other extracts such as hypaphorine (Hy), which is extracted from marine source, had been also shown to exert anti-inflammatory properties by inhibiting TNF-α, interleukin-1β (IL-1β), MCP-1 and vascular cellular adhesion molecule-1 (VCAM-1) in human mammary epithelial cells (HMECs). Thus, it could be considered as one of a potential alternative therapy in inhibiting inflammatory diseases, although the underlying molecular mechanism involving Hy remains unclear [52].

A study by Fardoun and colleague has successfully looked into the effect of a novel homoisoflavonoid known as 7-O-methylpunctatin (MP) on FBS-induced arteriolar SMC inflammation (microVSMCs), a model mimicking mild arteriolar inflammation. MP has been shown inhibited FBS-induced arteriolar SMC proliferation, migration, invasion and adhesion which aid in the ameliorating arteriolar inflammation in hypertension pathological conditions. Most instances of the utilisation of plant extracts were established in in vitro, which would certainly need to be further analysed along with in vivo before application in clinical studies. This VSMCs proliferation and migration inhibition hallmarks by those extracts would confer anti-inflammatory effects in mitigating intimal hyperplasia.

### 4.2. Genetic Manipulation and Protein Inhibition to Attenuate the Progression of IH

Other manipulation also can be performed by targeting a specific gene at the molecular level, which could also reduce the inflammatory factors. This has been done by generating a knock-out mouse as a model to demonstrate the effect of specific protein deficiency in elucidating their role in vivo.

Recent studies by de Jong and co-workers [53] demonstrated that the deficiency of P300/CBP-associated factor (PCAF) has led to the reduction of inflammatory cytokines release in vitro. The PCAF inhibition also alleviated infiltration of leukocytes that caused arterial injury, thus attenuating the progression of intimal hyperplasia in PCAF-knock-out (PCAF-KO) mice. The research work successfully identified the potential role of the lysine acetyltransferase PCAF in promoting the inflammation post-arterial injury and enhancing the proliferation of vascular smooth muscle cells.

Another study by Schlosser and colleague produced Mfap4-deficient mice to investigate microfibrillar-associated protein 4 (Mfap4) function in intima formation in vivo. The Mfap4-deficient mice showed delayed intimal formation after 14-days of formation of carotid arteries ligation. Mfap4 is localised in the ECM arteries, which is secreted by VSMC. At the cellular level, Mfap4 induced phosphorylation of FAK and activated the downstream protein involved in focal adhesion. FAK also acted through PI3K pathways, which were involved in VSMC migration and proliferation. Therefore, deficient of Mfap4 inhibited the intimal hyperplasia formation.

In another set of studies by Choi and colleague (2017), microRNA-155 (miR-155) has been used to target 3’UTR of cGMP-dependent protein kinase 1 (PKG1) mRNA. PKG1 is one of the downstream proteins in nitric oxide pathway that play an important role in VSMC vasorelaxation and phenotype control by Ca^2+^ channel. Therefore, downregulation of PKG1 by miR-155 implicates the Ca^2+^ regulation in the maintenance of VSMC vasorelaxation. The miR-155 also has been shown to enhance neointima formation through the autocrine and paracrine effects of smooth muscle-like cell-derived RANTES and thus can be targeted for neointima inhibition.

### 4.3. Targeted Drugs in Clinical Setting to Suppress IH

Pharmacotherapy targeted at different steps of the pathogenesis of neointimal hyperplasia can be used to prevent and treat this condition, specifically to block SMCs migration and proliferation. Currently, there are two types of drugs eluting stents that have been used, everolimus-eluting stents (EES) and paclitaxel-eluting stents (PES). These drugs block SMC migration and proliferation.

In a previous study by Sanders and co-workers [57], the utilization of anti-inflammatory and anti-proliferative agents, mainly both rosiglitazone (ROS) and pioglitazone (PGZ), has a significant effect in decreasing inflammation and cell proliferation and being able to supply a longer drug delivery to the adjoining tissue. The glitazone drugs, primarily ROS and PGZ, possess anti-inflammatory effects, which were used to induce adipose tissue to produce the vascular protective protein such as adiponectin. Their aim was to characterize the efficacy of ROS or PGZ combined with fat in vitro and in vivo. They also evaluated the feasibility of fat integrated with PGZ storage in decreasing intimal hyperplasia development in animal model. This novel approach of utilizing anti-inflammatory agents could possibly be considered as an alternative therapy to suppress arterial stenosis caused by intimal hyperplasia [57].

### 4.4. Emerging Role of Chemokines and Cytokines Induction to Combat IH/Cardiovascular Disease

Previous studies have shown that the production of the pro-inflammatory cytokines and chemokines expedite the atherogenesis process by enhancing the secretion of adhesion molecules such as MCP-1 and fractalkine [66]. This finally will lead to early recruitment of both monocyte and lymphocyte in the intimal region [67]. The presence of these molecules thus acts as a biomarker of coronary artery injury [68]. In fact, these pro-inflammatory cytokines and chemokines have been regarded as predictors of chronic heart failure [69].

The efficacy of anti-inflammatory and pro-inflammatory chemokines and cytokines, as the initial prevention to cardiovascular disease, has been researched on. Kitagaki and colleague [70] demonstrated that tumour necrosis factor receptor 1 (TNFR1) antagonist treatment successfully alleviates the severity of inflammation in arteries, thus attenuating intimal hyperplasia in a mice model. TNFR1 is actively involved in arterial inflammation; however, the underlying mechanisms of this factor in inhibiting intimal hyperplasia is yet to be elucidated. This study offers a promising outcome, which suggests that the intraperitoneal injections of TNFR1 antagonist can alleviate the growth of smooth muscle cells and reduce arterial inflammation, by inhibiting nuclear factor kappa B (NF-κB). TNF-α is known to possess a pro-inflammatory property that actively participates in immunity. TNF-α might also play a major role in arterial inflammation, which contributes to intimal hyperplasia [71,72].

Inflammation response is one of the mechanisms of action by our body against detrimental things such as infections, toxins and injuries, as an attempt to heal itself. In this case, damaged endothelial layer especially during vascular intervention has caused the formation of intimal hyperplasia. In this event, pro-inflammatory factors such as TNF-α and MCP-1 are released. This is what most of the current studies focused on by inhibiting pro-inflammatory factors without considering the equilibrium with anti-inflammatory factors and the consequences of the inhibition. On the other hand, researchers could enhance the expression of anti-inflammatory factors or fine-tune both expressions dynamically, which may compensate with other physiological changes. Incorporating biomaterial as a scaffold, inner and outer vessel could also reduce the formation of intimal hyperplasia by inhibiting the VSMCs migration and aneurysm, respectively. Therefore, complete information needs to be gathered to have a bird’s-eye view for halting the intimal hyperplasia development.

Different approaches can be performed to suppress intimal hyperplasia and heal the damaged lining according to the sequences of the intimal hyperplasia progression. At the early stages of damaged endothelial lining, induction of MCP-1 could be used to attract more monocytes recruiting on the damaged area. Therefore, external/additional MCP-1 protein can be introduced for the recruitment. Other cytokines or chemokines such as fractalkine (CX3CL1) can also be used to enhance the recruitment process. Fractalkine plays a major role in binding to CX3CR1 rapidly and firmly, which may directly contribute to monocyte tethering and arrest. In the meantime, introduction of 7-O-methyl punctatin (MP) as discussed previously can be used to halt the VSMCs migration and proliferation to avoid intimal thickening during the recruitment process. Once recruited, another induction can be used to induce the recruited monocyte to differentiate into macrophages and later differentiate into endothelial-like cells. Induction factors such as GM-CSF, VEGF and PDGF could be used to achieve the endothelial cell differentiation. While using the MCP-1, a pro-inflammatory factor, as a monocyte attraction, Ganoderma triterpenoids (GT) extracts could also be used as it has an anti-atherogenic effects in terminating inflammation in vein. GT could be used to buffer the reaction of MCP-1 in case it is overexpressed. Combination approaches like MCP-1, fractalkine, MP, differentiation factors and GT will eventually heal the damaged lining.

## 5. Conclusions

Intimal hyperplasia formation involves numerous cellular and molecular components. From this review, the utilisation of various factors effective in halting the formation of intimal hyperplasia, solely. However, their effectiveness as a whole has yet to be elucidated as researchers have only focused on single factors to combat this event. Combining different types of methods could possibly enhance the effectiveness of inhibition of intimal hyperplasia formation prior to being conducted in clinical trial. Despite the significant effects proven previously, there are several important aspects that shall be noted. Upon initiation of any inflammatory-associated research, the selection of cells, medium formulation, immunomodulatory agents and the subject used for proof-of-concept should be thoroughly discussed. These elements of selection are essential as they may influence the outcome of the study, which eventually could have a great impact on translating the scientific findings into medical therapies.

## Figures and Tables

**Table 1:** Summary of the 4 articles conducted in vitro that were selected from both Pubmed and Scopus databases.

No.	Authors	Type of Cells Used	Type of Cytokines/Chemokines/Extract/Substances/Scaffolds Used	Type of Disease	Methodology	Results	Conclusion
1	Choi et al., 2018 [49]	Vascular smooth muscle cell	TNF-α and IL-1β	Atherosclerosis, hypertension, stroke	VSMC was culture, treated and subjected to reporter gene assay, immunofluorescence, wound healing, proliferation, RTPCR, vascular tension assay and FACs analysis	TNF-alpha down-regulates PKG1 expression PKG1 suppression interceded by TNF-a was controlled by NF-kB NF-kB–responsive miR-155-5p has a direct aim on PKG1 VSMC induced by TNF-a moderated the expression of PKG1 The inhibition of PKG1 imitated the effect of TNF-a in interceding the alteration of VSMC phenotype PKG1 suppression reduced cGMP that moderate the vascular relaxation The stimulation of NF-kB plays a vital role in preventing the vasodilation disorder	The discovery elucidated the explicit role of inflammatory agents such as TNF-a in a pathological process of hypertension and atherosclerosis, which possibly caused by VSMC disorder through certain pathways. Thus, the signalling pathways involved is essential in determining the pathogenesis of other vascular diseases
2	Fardoun et.al, 2019 [50]	Humanvascular smooth muscle cells	7-O-Methyl Punctatin (MP)	Cardio-vasculo-pathologies (hypertension)	VSMC were isolated from redundant tissue of a newborn baby. The cells are then treated with MP MP was extracted, characterized, and purified prior to use Cells were subjected to BrdU assay, cell cycle analysis, RTPCR, SEM, wound healing, invasion and cell adhesion assay, monocyte adhesion assay, luciferase reporter assay and western blot	The cell proliferation of VSMC was suppressed by MP VSMC cell cycle was reduced by MP Cyclin D1 and CDK4 expression were attenuated, while CDK inhibitors, p21 and p27 were upregulated in VSMC, induced by MP. VSMC apoptosis were mediated by MP MP decreased cell migration and adhesion in VSMC MP reduced the expression of MMP-2 and MMP-9 in VSMC MP upregulated differentiation markers and downregulated the de-differentiation marker MP downregulated the expression of transcription factor, NF-κB and suppressed the initiation of its inhibitor	These findings showed that MP could be treated as a regulator in cardio-vasculo pathologies, by attenuating the alteration in VSMC phenotype
3	Kenagy et al., 2016 [51]	Human saphenous vein cell lines	1. Platelet-derived growth factor (PDGF)-BB 2. Collagen gel	Intimal hyperplasia	Human saphenous vein cell lines used was subjected to gene expression analysis Cells were stimulated with PDGF-BB or serum within 4 h The regulation of gene expression was evaluated by Patterns of Gene Expression software Cell migration was performed using a microchemotaxis chamber to investigate the effects of PDGF-BB Collagen gel contraction, which was used as a model of vascular remodelling, was determined using bovine collagen Bovine collagen gel has been used for vaso-remodelling	Migration in response to PDGF-BB showed no correlation with graft outcome The graft outcome has not correlated with cell migration treated with PDGF-BB Cell migration induced by PDGF-BB was not affected by heparin Cell treated with FBS showed the expression of 1188 unique genes, whereas cell treated with PDGF expressed 1340 genes Sky-blue modules—No significant enrichment in GO terms after treatment with FBS. Yellow modules—No significant enrichment in Go terms after treatment with PDGF Bovine gel contraction—No significant effects was noticed upon SCARA5 knockdown, whereas SBSN knockdown improved the contraction Treatment with PDGF-BB showed that the expression of SCARA5 was escalated, but no significant effect can be seen for SBSN Proliferation assay—No significant effect on SBSN knockdown. SCARA5 knockdown showed slight increment in cell proliferation	Elucidation of SCARA5 and SBSN illustrated that these genes has a great effect for the future therapy of vein graft impairment
4	Sun et al., 2017 [52]	Human micro-vascular endothelial cells (HMEC-1)	Hypaphorine (Hy) derived marine	Vascular endothelial dysfunction and atherosclerosis	HMEC-1 was cultivated in MCDB 131 medium with 10% FBS Human EA. hy926 endothelial cells were cultivated in DMEM with 10% FBS until confluent The concentration of Hy inhibitors were selected based on optimization that has been reported previously Parameters: RTPCR, western blot, ICC, siRNA transfections, ELISA	TNF-α, IL-1β, VCAM-1 and MCP-1 were upregulated at 500 ng/mL of Hy, and at higher dose (100 µM) attenuates the expression of these genes. TLR4 inhibition affected Hy inflammatory mechanism PPAR-γ interceded in vascular protection of Hy in HMEC-1 PI3K/Akt/mTOR inhibitor plays a significant effect in HMEC-1 upon inflammation, as showed by negative correlation between PPAR-γ and TLR4	Hy plays a major role in the anti-inflammatory effect of HMEC-1 via the activation of PPAR-γ and TLR4. Thus, it could potentially act as an alternative anti-inflammatory factor for the treatment of inflammatory-related diseases

**Table 2:** Summary of the 8 articles conducted in vitro and in vivo that were selected from both Pubmed and Scopus databases.

No.	Authors	Type of Cells/Animals Used	Type of Cytokines/Chemokines/Extract/Substances/Scaffolds Used	Type of Disease	Methodology	Results	Conclusion
1	de Jong et al., 2017 [53]	In vitro: 1. Vascular smooth muscle cells (VSMC) from PCAF KO aorta 2. Bone-marrow derived macrophages from PCAF KO bone marrow 3. Whole blood-derived leukocytes from PCAF KO blood In vivo: PCAF KO transgenic male mice	Garcinol on PCAF KO mice (Genetic P300/CBP-associated factor	Intimal hyperlasia	Chow diet were fed to PCAF KO and WT, whereas ApoE3-Leiden mice were given a Western diet consisted a mixture of cholesterol and cholate to promote hypercholesterolemia in animal model WT and PCAF KO were subjected to vascular inflammation via femoral artery cuff 10 μL pluronic gel F127 ± 25 mg/mL garcinol treatment was given to ApoE3-Leiden mice. Garcinol was applied for few days at the inflammatory site Parameters: Cell viability, IHC, morphometric analysis and ELISA	The lack of PCAF attenuates the production of inflammatory cytokine PCAF KO mice showed the reduction of intimal hyperplasia VSMC, leukocytes and macrophages showed the downregulation in CCL2, IL-6 and TNF-a Macrophage influx and CCL2 regulation were not affected by PCAF deficiency Inflammatory cytokines were inhibited by garcinol treatment PCAF inhibitory effect decreased injury in vivo	The finding defined an essential contribution of lysine acetyltransferase PCAF in regulating inflammatory effect associated to intimal hyperplasia
2	Haiming et al., 2017 [54]	In vivo: New Zealand white rabbits	Collagen external scaffold (CES)	Intimal hyperplasia	New Zealand white rabbits (n=36) were divided into several groups: (a) Without graft (b) With graft (c) CES All rabbits were subjected to AVFs The AVFs was encircled by CES. The parameters of AVFs were evaluated during surgery and ultrasonic evaluation was performed after 4 weeks of surgery, followed by histological analysis of vein grafts. mRNA level and protein expression were quantified using qPCR and Western blot analysis. The markers used are as follows: (a) Proliferating cell nuclear antigen (PCNA) (b) Active cleaved-caspase-3 (ClvCasp-3) (c) Smooth muscle 22 alpha (SM22-alpha)	CES successfully prevented the growth of vein grafts by the alteration of diameter Decrease of blood flow in vein graft was impeded effectively by CES Intimal thickening was decreased by CES and significantly ameliorated the restoration of vein graft The expressions of PCNA and ClvCasp-3 were downregulated, and the expression of SM22-alpha was upregulated by CES	CES provided a significant role in reducing the formation of intimal hyperplasia. It also had a useful impact in ameliorating the vein graft remodelling
3	Hsu et al., 2018 [55]	In vitro: Human umbilical vein endothelial cells In vivo: Carotid artery-ligation mouse model (Male BALB/c)	Ganoderma triterpenoids (GT) extracted from *Ganoderma lucidum* (mushroom)	Neointimal hyperplasia, atherosclerosis	Mouse model was subjected to GL and GT treatment that promote atherogenesis GL and GT treatment on neointima formation induced mouse (by a ligature at the end of carotid artery (LCA)) HUVECs were seeded on gelatine-coated plates in medium supplemented with 10% FBS, heparin and 30 μg/mL endothelial cell growth supplement HUVECs were cultivated on plates coated with gelatine. The cells were cultured in a medium supplemented 10% FBS, heparin with additional 30 μg/mL endothelial cell growth factor Pre-treatment of GTs (500 μg/mL) or DMSO vehicle control were given for 1 h prior to treatment with H_2_O_2_ (400 μM) that was applied. It was incubated for another 24 h Parameters: Cell characterization by flowcytometry, reactive oxygen species, immunofluorescence and in vitro perfusion system	GL treatment prevented carotid artery from atherogenesis derived flow system GT treatment protected carotid artery from neointima formation GT reduced the oxidative stress and atherogenic events in HUVECs GTs inhibit the fluctuation of flow system-induced inflammation in HUVECs GTs provide a protection in HUVECs against oxidative stress GT treatment successfully halting atherogenesis	Anti-atherogenic effects provided by GL and GT are essential in terminating inflammation of HUVECs, which was promoted by disturbed flow system
4	Liu et al., 2016 [56]	In vitro: Primary foetal fibroblasts from black pig (as donor cells) In vivo: Pigs (undergone somatic cell nuclear transfer (SCNT) and embryo transfer) to induced hfat-1 transgenic pigs	Not applicable	Cardiovascular disease	The hfat-1 plasmid was transfected into primary foetal fibroblasts. The positive colonies were served as donor cells to conduct SCNT and embryo transfer into pig model. The pigs were housed in 18–20 °C environment. The diets were given three times daily, with a mixture of 15% crude protein and 4 % crude fat Genomic DNA derived piglets were isolated from tails, followed by gene expression analysis (PCR) and subsequently by gel electrophoresis and gene sequencing GC–MS was performed to evaluate the constituents of fatty acid in the ears based on previous preliminary studies 5ml of blood were collected from pig vein following 16 h of food depreciation. Beckman coulter UniCel DxC 800 Synchron was used to determine the level of TAG, total cholesterol (TC), HDL-C and LDL-C	PCR analysis showed positive expression of hfat-1 transgene in all piglets, which proved by the accumulation of n-3 PUFAs The expression of n-3 PUFAs is 2-fold higher in transgenic pigs compared to wild-type pigs There were no significant changes of n-6 PUFAs in transgenic pigs, and the level composition of fatty acids was alleviated in transgenic pigs compared to wild type pigs No significant different of total cholesterol, HDL-C and LDL-C levels in both type of pigs in deprivation condition, whereas TAG level was notably attenuated in transgenic pigs compared to wild-type pigs The transgenic pigs showed downregulation of MCP-1, IL-6 and TNF-α compared to wild-type pigs, which demonstrated that the accumulation of n-3 PUFAs plays a positive role in reducing the inflammatory effects	These findings revealed that the aggregation of n-3 PUFAs provides a promising impact on vasculoprotective studies. The use of transgenic pigs might be beneficial in determining the inflammatory mechanisms in the body
5	Sanders et al., 2017 [57]	In vitro: Human venous smooth muscle cells In vivo: 1. Porcine model 2. Adipocytes 3. Peripheral blood mononuclear cells	1. Rosiglitazone drugs (ROS) 2. Pioglitazone drugs (PGZ)	Stenosis caused by intimal hyperplasia (Develops following the installation of arterial synthetic grafts during haemodialysis)	ROS or PGZ in powder form (6–6000 μM) was integrated thoroughly with adipose tissue explants and cultivated in the medium HPLC/MS/MS was performed to quantify the drug that release from adipose tissue. Secretion of adiponectin and monocyte chemotactic protein-1 (MCP-1) in the conditioned media were determined by ELISA. Platelet-derived growth factor-BB (PDGG-BB) was evaluated through DNA binding assay, whereas lipopolysaccharide (LPS) was measured by ELISA HPLC/MS/MS were also performed to determine the kinetics of PGZ. MRI were used to monitor the depository of adipose in a porcine model The effects of PGZ/ fat depository were assessed using a porcine model with the installation of synthetic graft at the region between carotid artery and jugular vein	Powdered PGZ and ROS induced the secretion of adiponectin in conditioned media The secretion of MCP-1 in conditioned media were increased. However, the treatment using PGZ and ROS alleviated MCP-1 level Drugs was not detected in the pig treated with fat/PGZ depot but slight detection (0.06 ± 0.06 μM) can be seen in the pig with fat/ROS depots treatment. MRI monitoring demonstrated that the PGZ/fat depots resided near the perivascular region of jugular vein The expression of adiponectin protein in jugular vein was increased in the fat/PGZ transplant Treatment with fat/PGZ depots showed identical graft lumen compared to control group	Treatment with fat/PGZ depots effectively regulates the expression of adiponectin and reduced inflammatory activities. Hence, the findings could be considered as a promising therapy in halting stenosis due to intimal hyperplasia
6	Schlosser et al., 2015 [58]	In vitro: Vascular smooth muscle cells and monocyte In vivo: *Mfap4*-deficient (*Mfap4−/−*) mice Ex vivo: Neointima of *Mfap4*-deficient (*Mfap4−/−*) mice	Microfibrillar-associated protein 4 (MFAP4)	Neointimal hyperplasia	Materials and Methods were described in data supplement that are available online	Mfap4−/− mice impeded neointima development following artery ligation. This is related to the proliferation of VSMC and the invasion of leukocyte The presence of MFAP4 was detected both in healthy and diseased vessel walls, thus stimulating the development of neointima VSMC synthesized MFAP4 and mediated binding to integrin αVβ3 Stimulation MFAP4 mediated cell proliferation and induced the migratory effect on VSMC MFAP4 is effective in protecting VSMC from apoptosis and monocytic cell migration	MFAP4 has a great effect in regulating integrin αVβ which promoted VSMC migration and proliferation, thus increased the formation of neointimal hyperplasia
7	Wang et al., 2018 [59]	In vitro: Smooth muscle-like cells (SMLCs) from the neointima of the outflow vein In vivo: 1. C57BL/6 mice 2. miR-155^-/-^ mice (C57BL/6)	Not applicable	Neointimal hyperplasia	C57BL/6 and miR-155 mice were subjected to AVFs at the region of jugular vein and carotid artery The mice were euthanized. At days 7, 14, 21 and 28, the segments were harvested. The AVFs were subjected to histological staining, and gene expressions were evaluated using RTPCR Native saphenous vein was obtained from patients who underwent CABG surgery and suffered from chronic kidney failure. The samples were assessed by histological staining	Knockout of miR-155 decreases the level of proinflammatory cytokines in the outflow vein and attenuates the formation of neointimal hyperplasia Knockout of miR-155 halted SMLCs proliferation and alleviates the regulation of ECM RANTES was upregulated in SMLCs with the increment of miR-155 SMLCs proliferation and ECM expression were enhanced by miR-155 due to high level of RANTES Upregulation of RANTES expression was promoted by miR-155 Patients with neointimal hyperplasia showed the high expression of inflammatory factors in the outflow vein	miR-155 promotes the formation of intimal hyperplasia through the upregulation of RANTES that provide inflammatory effects in SMLCs
8	Yang et al., 2016 [60]	In vitro: Adipose tissue–derived mesenchymal stem cells (MSC) In vivo: B6.Cg-Foxn1nu/J mice	Not applicable	Neointimal hyperplasia	B6. Cg-Foxn1nu/J mice were subjected to arteriovenous fistula (AVF) and utilized for the experiment MSCs was labelled with GFP and directly injected into outflow vein upon AVF installation At day 7, mice were euthanized following AVF installation. The tissues were analysed by RTPCR and histomorphometric analyses. At day 21, the tissues were evaluated only by histomorphometric analysis. MSCs labelled with 89Zr were implanted in a different group. The animals were examined using positron electron tomography for 3 weeks. The data were analysed using two-way analysis of variance and subsequently by Student t test	Transplanted MSCs showed GFP-positive cells that were detected at day 7, whereas at day 21, GFP signal cannot be seen MCP-1 was downregulated at the outflow vein resulted from MSCs transplantation Transplanted MSCs attenuates the neointimal, medial and adventitial regions, and cell concentration was also decreased, whereas the lumen area was increased at day 7 and day 21 TUNEL staining demonstrated positive staining at the outflow vein resulted from MSCs transplantation MSCs transplantation alleviates cell proliferation and decreases the expression of FSP-1, a-SMA, HIF-1a and CD68	Adventitial transplantation of MSCs decreases MCP-1 gene expression, accompanied by a reduction in venous neointimal hyperplasia MSCs transplantation reduced the expression of MCP-1, and thus reduced the formation of neointimal hyperplasia
